# LCN2: Potential Targets for Cancer via Ferroptosis-Related Pathways

**DOI:** 10.32604/or.2026.090752

**Published:** 2026-10-28

**Authors:** Juan Zhang, Ying Chen

**Affiliations:** Department of Gynecology, The Fourth Affiliated Hospital of China Medical University, Shenyang, China

**Keywords:** Lipocalin 2 (LCN2), ferroptosis, tumor microenvironment, biomarker, cancer therapy

## Abstract

Lipocalin 2 (LCN2) was initially identified as a lipid carrier protein, and recent studies have revealed it acts as a multifunctional regulator of cancer progression that is closely associated with ferroptosis. Ferroptosis is a newly discovered form of programmed cell death, characterized by alterations in intracellular iron content and lipid peroxidation levels, and disruption of antioxidant defense systems. Given the close connection between ferroptosis and multiple diseases, particularly cancer, investigation into the mechanisms underlying ferroptosis has become a key research direction in recent years. Recently, a growing body of research has demonstrated that LCN2 plays important roles in ferroptosis regulation and exhibits context-dependent effects across different malignant tumors, while the molecular basis behind this phenomenon remains not fully understood. Given these findings, LCN2 is considered a promising therapeutic target for tumor progression. Accordingly, this review summarize how LCN2 interacts with molecules in ferroptosis-related pathways, its diverse roles within the same cancer, and its regulatory functions in the tumor microenvironment. Additionally, this review discusses the current clinical application of LCN2 as a biomarker and therapeutic target, Thus, this review aims to elucidate the role of LCN2 in cancer progression and its immense potential as a therapeutic target. It is hoped that these research findings will be translated into clinical cancer treatment strategies.

## 1 Introduction

Lipocalin 2 (LCN2), also known as neutrophil gelatinase-associated lipocalin (NGAL), is a secreted member of the lipocalin superfamily that has emerged as a multifunctional regulator involved in iron metabolism, inflammation, and cancer progression [1,2]. At first, LCN2 identified as an early diagnostic marker for acute kidney injury (AKI), with its serum levels demonstrating high predictive value for postoperative AKI [3]. Since its discovery, more than 70 clinical trials worldwide have incorporated LCN2 into diagnostic and prognostic assessment systems [4]. Over the past decade, the role of LCN2 in tumor biology has become increasingly prominent: studies have shown that LCN2 acts as an oncogene that promotes the progression of breast cancer [5], myeloproliferative neoplasm [6], skin squamous cell carcinoma [7], gastric carcinoma [8], thyroid cancer [9], where it is frequently associated with enhanced tumor proliferation, invasion, metastasis, and therapeutic resistance. Nevertheless, some studies have found that, for example, in leukemia, low LCN2 expression is associated with a poor prognosis [10]; In prostate cancer tissue with bone metastases, elevated LCN2 expression can inhibit the growth of prostate cancer cells, thereby improving patients’ chances of survival [11]. In conclusion, unlike classical oncogenic factors, the biological function of LCN2 appears to be highly context-dependent, exhibiting both tumor-promoting and tumor-suppressive properties depending on tumor type, genetic background, and microenvironmental conditions.

Recently, the role of LCN2 in iron homeostasis and ferroptosis regulation has recently emerged as a central point in contemporary cancer research. As an essential metal ion for the human body, iron is indispensable for a myriad of critical cellular processes, including oxygen transport (via haemoglobin synthesis), cell cycle progression, DNA replication and repair, oxidative phosphorylation, and tricarboxylic acid (TCA) cycle metabolism [12]. However, excessive intracellular iron also can lead to cell death or promote genetic mutations and tumorigenesis, so the iron supply is limited in human body: iron intake, transport, and excretion are strictly regulated. Studies have shown that iron homeostasis dysregulation is closely associated with the pathogenesis of malignant tumors: cancer cells enhance iron uptake by upregulating the expression of the transferrin receptor (TFRC) [13], activate NCOA4-mediated ferritin autophagy to promote the release of stored iron [14], and downregulate the iron efflux protein FPN1 to reduce iron efflux, ultimately creating a state of intracellular iron overload to meet the iron demands of rapid proliferation [15]. This abnormality in iron metabolism is not only a central feature of tumor metabolic reprogramming, but also a key driver of tumor initiation, proliferation, invasion, and treatment resistance.

Ferroptosis is an iron-dependent form of programmed cell death formally named by Dixon et al. in 2012 [16]. Its core mechanisms involve the disruption of cell membrane integrity due to intracellular iron accumulation, excessive production of lipid reactive oxygen species (ROS), and the peroxidation of polyunsaturated fatty acids (PUFAs), as well as the dysregulation of the glutathione peroxidase 4 (GPX4)-glutathione (GSH) antioxidant defense system [17]. As mentioned before, tumor cells must maintain high iron uptake to meet their high proliferative demands. But excessive intracellular iron simultaneously catalyzes the formation of ROS via Fenton chemistry, thereby promoting ferroptosis, so tumor cells probably are more susceptible to ferroptosis than normal cells [18,19]. This dual role creates a biological paradox in which cancer cells exhibit both dependence on iron and vulnerability to iron-induced cytotoxicity, which may represent a therapeutic window for future ferroptosis-targeted interventions. Emerging studies suggest that LCN2 represents a previously underappreciated regulator connecting iron metabolism with ferroptosis in cancer. In the majority of solid malignancies, LCN2 acts as a tumor-promoting factor by regulating iron homeostasis and suppressing ferroptosis. Conversely, other studies have suggested that LCN2 acts as a tumor suppressor, for example, in gastric cancer, LCN2 has been reported to promote ferroptosis [20].

Recent advances have further expanded the biological significance of the LCN2–ferroptosis axis beyond tumor cells themselves. The tumor microenvironment (TME) represents a dynamic ecosystem in which iron distribution and metabolic communication critically influence cancer progression. Immune cells, particularly tumor-associated macrophages and neutrophils, can secrete LCN2 to reshape iron availability within the TME, thereby affecting tumor growth, immune evasion, and therapeutic resistance [21,22]. Thus, LCN2 may function not only as an intracellular regulator of ferroptosis but also as an extracellular mediator coordinating iron redistribution between different cellular compartments within tumors.

Given the emerging importance of iron metabolism and ferroptosis in cancer therapy, understanding how LCN2 integrates iron homeostasis, ferroptosis, and TME interactions may provide new insights into cancer biology and therapeutic development. In this review, we summarize the molecular mechanisms underlying LCN2-mediated regulation of iron metabolism, discuss its context-dependent roles in promoting or suppressing ferroptosis across different cancer types, and elucidate its functions in remodeling the TME. Furthermore, we examine the therapeutic potential of targeting the LCN2–ferroptosis axis and discuss future directions for exploiting LCN2 as a biomarker and therapeutic target in precision oncology. The purpose of this study is to summarize the role of LCN2 in ferroptosis in tumors to date, thereby elucidating how LCN2 influences tumor cell progression and providing new insights for cancer treatment.

## 2 The Molecular Basis of Iron in LCN2

LCN2, which was first isolated from rat kidney cells in 1989, belongs to the lipocalin superfamily with a molecular weight of approximately 25 kDa. Subsequently, Kjeldsen et al. Isolated and determined the complete primary structure of NGAL from human neutrophils in 1993, and confirmed that NGAL was a secretory peptide composed of 198 amino acid residues. All members of the lipocalin superfamily share a common tertiary structure feature, called the ‘the lipocalin fold’. The lipocalin fold consists of eight antiparallel β sheets, bonded throughout the fold with hydrogen bonds to one another, thereby making a β-barrel. The cavity of LCN2 is broader and shallower than that of other proteins in the same family, allowing it to bind and transport small hydrophobic ligands (including siderophore) [1,23,24].

In 2002, Goetz and colleagues discovered that LCN2 functions as an iron-binding protein [23], laying the molecular foundation for its subsequent role in the regulation of iron metabolism. Subsequent studies have shown that LCN2 can specifically bind to and sequester the bacterial iron carrier enterobactin (Ent), which has an extremely high affinity for iron, surpassing that of most iron-binding proteins in the host [25], thereby preventing pathogenic bacteria from taking up iron from the host and exerting an antibacterial effect. In addition, other studies further revealed that LCN2 can also bind to endogenous mammalian siderophores (like catechol), participating in host iron transport processes [26]. These studies reveal that LCN2 is not a simple lipid carrier, but a secreted protein with dual functions of iron binding and iron transport. The biological characteristics of LCN2 have become more complex due to its structural heterogeneity and differences between different species. LCN2 in humans can be a 25 kDa monomer, a 45 kDa homodimer, and a 135 kDa heterodimer with MMP9 (92 kDa) [27]. The monomeric form is an effective iron chelator, while the dimeric form loses its iron binding ability. In contrast, MMP-9 cannot form heterodimers in mouse models [28]; This species difference poses a certain obstacle to the translational development of LCN2 targeted therapies (see Section 6 for details).

Based on the iron binding state, LCN2 can be divided into two functional forms in structure: iron-free LCN2 (apo-LCN2) and iron-bound LCN2 (holo-LCN2) [25]. Notably, in cells expressing 24p3R (a receptor with high affinity for LCN2), holo-lCN2 enters cells through this receptor, releasing iron ions to increase intracellular iron storage; Conversely, apo-LCN2 binds to intracellular siderophores to chelate iron and transport it to the extracellular environment, thereby reducing intracellular iron concentrations. In addition, apo-LCN2 also can upregulate the expression of the pro-apoptotic molecule Bim, ultimately inducing apoptosis [29,30]. Therefore, apo-LCN2 and holo-LCN2 achieve opposite directions of iron transport, constituting a precise regulatory mechanism for intracellular iron homeostasis (Fig. 1). Under physiological conditions, most LCN2 in the circulation exists in the apo-LCN2 form; when inflammation or infection occurs, bacteria produce large amounts of siderophores, and apo-LCN2 binds to iron carrier-iron complexes to convert to holo-LCN2, thereby exerting antibacterial and iron transport functions [31].

**Figure 1:**
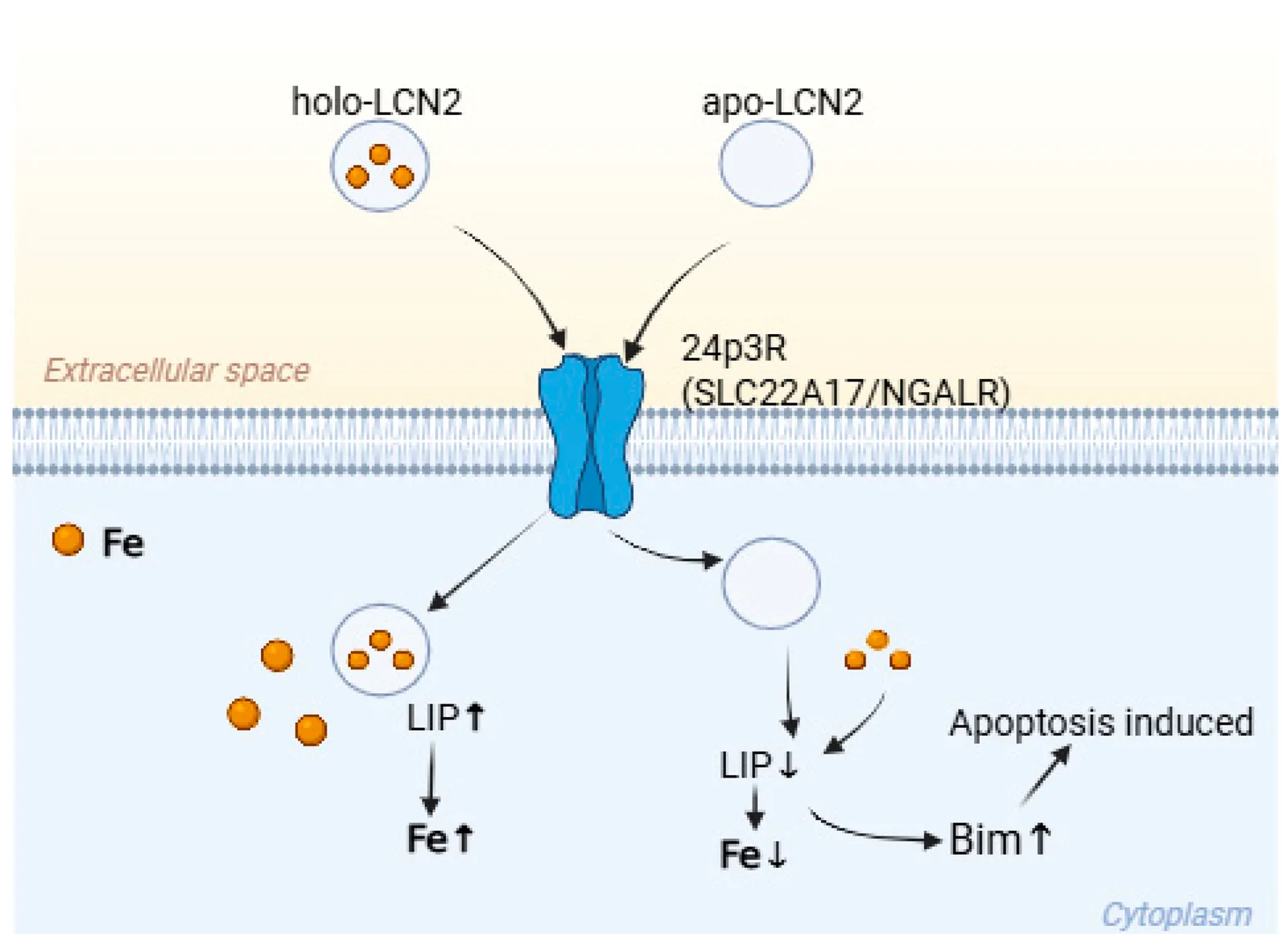
The “iron transport switch” mechanism of Iron bound LCN2 (holo-LCN2) and Iron free LCN2 (apo-LCN2). In the context of 24p3 receptor (24p3R), different structures of lipocalin 2 (LCN2) only exert opposite effects: holo-LCN2 enters the cell through 24p3R, increasing the concentration of iron ions in the labile iron pool (LIP); After entering the cell, iron free LCN2 apo-LCN binds to iron ions, causing a decrease in iron ion concentration; Simultaneously activate Bim protein and induce cell apoptosis. The figure was prepared using BioRender.com.

However, in most tumor cells, although LCN2 has a dual function, its main function is as a ferroptosis inhibitor. The functional dimorphism of LCN2 may stem from its two molecular states (apo-LCN2 and holo-LCN2), tumor type, or differential expression of receptors. In theory, apo-LCN2 actively outputs intracellular iron through 24p3R mediated endocytosis and recycling of endosomes, which may promote cell apoptosis and even ferroptosis. However, existing evidence suggests that in most solid malignant tumors, LCN2 mainly protects tumor cells from ferroptosis by interacting with ferroptosis related pathways or cytokines, or by affecting antioxidant pathways (see Section 3 for details). The relative contribution of apo-LCN2 and holo-LCN2 to the fate of tumor cells and the regulatory mechanism of their mutual transformation in the TME still need to be fully elucidated. Further research is needed in the future to address these issues. In the following chapters, we will summarize our current understanding of how LCN2 interacts with ferroptosis related signaling molecules and reshapes iron homeostasis in the TME.

## 3 Roles of LCN2 in Ferroptosis Pathways

Various published studies have shown that LCN2 plays a key role in the process of ferroptosis. It participates in regulating ferroptosis in tumor cells through various mechanisms. Notably, LCN2 has a dual role in tumors: In most cases, LCN2 molecules act as oncogenes, inhibiting ferroptosis in tumor cells, while others act as tumor suppressors, promoting ferroptosis in tumor cells. This may be influenced by multiple signaling pathways in its expression and function, and its overall impact on cancer progression and treatment response is highly context dependent.

### 3.1 The Interactions between LCN2 and Various Ferroptosis Signaling Molecules and Pathways

Cancer is an area where LCN2 plays an important role. It can interact with multiple signaling pathways, and transcription factors to affect the duration of ferroptosis. These pathways play a role in different types of tumors, and even play different roles in the same type of tumor. This chapter will explore these aspects in detail (Fig. 2).

In most malignant tumors, LCN2 suppresses the ferroptosis process by interacting with various signaling pathways. Nuclear factor kappa-B (NF-κB) is an important transcription factor reported to regulate LCN2 expression, which is considered a key factor in nearly all cancers [32]. For example, Villin-1 (VIL1), a cytoskeletal protein in intestinal epithelial cells, can bind to and activates the NF-κB pathway, thereby upregulating LCN2 expression and inhibiting ferroptosis; knocking out VIL1 significantly reduces LCN2 expression, restores the sensitivity of colorectal cancer cells to ferroptosis, and inhibits tumor growth *in vivo* [33]. Similarly, in glioblastoma, activation of the NF-κB pathway promotes transcription and expression of the LCN2 gene, leading to a decrease in intracellular iron content and a weakening of lipid peroxidation. At the same time, it also develops resistance to the chemotherapy drug temozolomide, which is achieved by evading the ferroptosis mechanism [34].

On the contrary, other evidence indicates that loss of LCN2 activates the NF-κB pathway in colorectal cancer, leading to increased ferroportin (FPN) expression, enhanced iron efflux, reduced Fenton chemistry, and ultimately attenuated cisplatin-induced ferroptosis, thereby conferring cisplatin resistance [35]. Similarly, gastric cancer represents another an important example in which LCN2 exhibits a ferroptosis-promoting function. The tumour suppressor WTX-L activates the NF-κB pathway to upregulate LCN2, which drives ferroptosis through iron overload and lipid peroxidation [20] (Fig. 3).

Additionally, in hepatocellular carcinoma, Signal Transducer and Activator of Transcription 3 (STAT3) activates the major vault protein (MVP), an oncogenicRNA-binding proteins, could then bind to and maintain the stability of LCN2, which depletes iron and increases the insensitivity of HCC (hepatocellular carcinoma) to ferroptosis inducers, simultaneously mediated resistance to Sorafenib [36].

Additionally, nuclear protein 1 (NUPR1) is a transcription factor that directly influences LCN2 expression. In colorectal cancer, NUPR1 directly binds to the LCN2 promoter and induces LCN2 expression, resulting in decreased intracellular iron accumulation, reduced lipid peroxidation, and enhanced resistance to ferroptosis [37]. Similarly, CDX2-driven activation of NUPR1 promotes LCN2 expression and contributes to ferroptosis resistance in stage II–III colorectal cancer [38]. Moreover, in pancreatic ductal adenocarcinoma (PDAC), NUPR1 also functions as a ferroptosis-suppressive regulator by increasing LCN2 expression, reducing intracellular iron accumulation, and protecting tumor cells from ferroptotic stress [39]. In an aging-related lung cancer model, NUPR1 directly binds to the LCN2 promoter to induce high LCN2 expression. LCN2 chelates free iron in the cytoplasm, causing functional iron deficiency in cells and reducing the amount of active iron available for the Fenton reaction, thereby decreasing sensitivity to ferroptosis [40]. In breast cancer, Wilms’ tumor-associated protein (WTAP) and glutamine synthetase (GS) can activate or regulate NUPR1 expression, thereby enhancing LCN2 expression. LCN2 then inhibits ferroptosis by sequestering or expelling iron, thereby promoting the proliferation, migration, and invasiveness of breast cancer cells, as well as cisplatin resistance [41,42].

P53 is also the core transcription factor of ferroptosis, and has two-way interaction with LCN2. Previous studies have found that p53, NF-κB and androgen receptor together constitute the upstream network controlling NGAL expression in prostate cancer cells [43]. On the other hand, in myeloproliferative tumors, LCN2 secreted by the tumor increases the level of iron in adjacent normal cells, resulting in the increase of intracellular ROS level, which is likely achieved by activating p53 pathway [44]. Under the background of the defect of p53 gene function, the expression level of LCN2 in oviduct epithelial tissue is increased, which activates NF-κB signaling pathway, thereby inhibiting ferroptosis [45].

In addition, Jiang et al. found that Forkhead box protein M1 (FOXM1) upregulates LCN2 by binding to its promoter, reducing malondialdehyde (MDA) and ROS levels, thereby promoting cisplatin resistance and inhibiting ferroptosis [46].

Therefore, the dual role of LCN2 in iron mediated cell death, even within the same pathway, may result in opposite effects, which may be attributed to differences in tumor types and the state of the TME. More research is needed in the future to explore its mechanism.

### 3.2 Activation of the Antioxidant Defense System

In addition to interacting with a variety of pathways, LCN2 also directly or indirectly affects the antioxidant system and lipid peroxidation process, thereby affecting the occurrence of ferroptosis (Fig. 2).

The Xc^−^ system, composed of SLC7A11 and SLC3A2, is responsible for the uptake of cystine, which serves as a substrate for glutathione (GSH) synthesis; GSH acts as a cofactor for GPX4, and together they protect cells from damage caused by various oxidative stress conditions [47]. In glioblastoma (GBM), LCN2 knockdown results in a significant decrease in GPX4 expression and increased sensitivity to ferroptosis inducers [48]. Cell-based experiments confirmed that LCN2 overexpression in colorectal cancer cells significantly upregulates the protein levels of GPX4 and xCT, ultimately inhibiting erastin and 5-FU-induced ferroptosis and conferring chemotherapy resistance [49]. Similarly, LCN2 also enhances or stabilizes the activity of XC^−^ transporter by stabilizing CD44 variants, and subsequently enhance the survival of ovarian clear cell carcinoma (CCC) cells [50]. Furthermore, a high-iron environment can induce increased LCN2 expression in normal human bronchial epithelial cells, inhibiting ferroptosis by enhancing upregulation of GPX4. However, this process may simultaneously promote malignant transformation [51]. Therefore, LCN2 can positively regulate the expression of GPX4 and GSH and inhibit lipid peroxidation.

Similarly, in cervical squamous cell carcinoma, TMPO-AS1, is a long non-coding RNA (lncRNA), protects LCN2 from degradation by directly binding to the LCN2 protein, thereby upregulating LCN2 levels and decreasing iron accumulation in tumor cells and upregulateing the expression of SLC7A11, which will reduce lipid peroxidation, leading to ferroptosis resistance, thereby promoting tumor growth and invasiveness [52].

**Figure 2:**
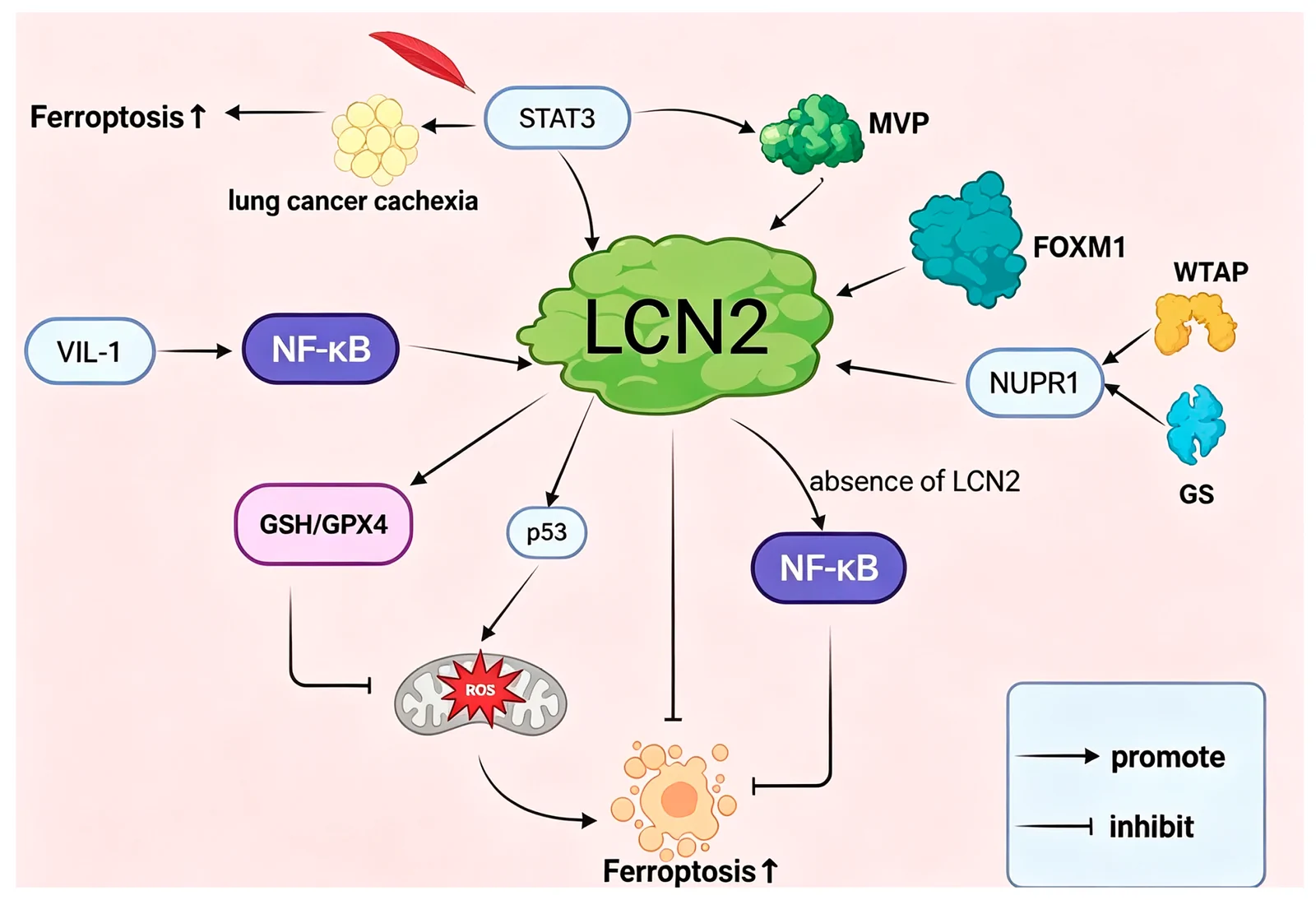
The Interactions between lipocalin 2 (LCN2) and Various Ferroptosis Signaling Molecules and Pathways: Various upstream signaling pathways, including regulatory networks associated with P53, Villin-1 (VIL-1), Signal Transducer and Activator of Transcription 3 (STAT3), nuclear factor kappa-B (NF-κB), major vault protein (MVP), Forkhead box protein M1 (FOXM1), and nuclear protein 1 (NUPR1) collectively regulate LCN2 expression. Subsequently, LCN2 influences ferroptosis by affecting antioxidant defense and oxidative stress or by directly regulating cellular susceptibility to ferroptosis. Conversely, the absence or downregulation of LCN2 may activates NF-κB signaling and inhibit ferroptosis. In the context of lung cancer cachexia, the STAT3–LCN2 axis may also be involved in ferroptosis processes associated with non-tumor cells. Taken together, these pathways reveal that LCN2 links upstream oncogenic signals to ferroptosis through a complex and context-dependent regulatory network. The figure was prepared using BioRender.com.

### 3.3 Different Effects of LCN2 on the Same Cancer

In most cancers, the overexpression of LCN2 gene mainly inhibits the ferroptosis process by capturing iron and inhibiting lipid peroxidation, thus promoting the survival, migration and resistance of tumor cells to chemotherapy drugs; However, in some cancers, such as lung cancer, renal cell carcinoma, colorectal cancer and acute lymphoblastic leukemia, the regulation of LCN2 on ferroptosis process is not unidirectional, but highly dependent on the specific situation.

In lung cancer, the expression level of LCN2 is too high and is associated with poor prognosis. Upregulation of LCN2 expression can inhibit ferroptosis by regulating iron homeostasis and lipid peroxidation, thereby promoting tumor proliferation and mediating gefitinib resistance [53,54,55]. However, in lung cancer cachexia, tissue-infiltrating neutrophils secrete LCN2 and release it throughout the body, acting on adipose tissue and skeletal muscle tissue, triggering ferroptosis by activating STAT3, thereby exacerbating metabolic depletion, weight loss, and the risk of death [22]. Notably, this systemic effect reflects ferroptosis in non-tumor tissues rather than a pro-ferroptotic role of LCN2 within lung cancer cells.

In renal cell carcinoma (RCC), mutations in the tumor suppressor von Hippel-Lindau (VHL) gene lead to increased production of LCN2, which causes oxidative stress and ferroptosis, ultimately restraining tumor growth [56] (Fig. 3). In contrast, holo-LCN2 can activate Nrf2 pathway (an important oxidative stress/antioxidant signaling pathway) and trigger the integrated stress response (ISR) reaction, which promotes ATF4 activation and increases the expression of ferroptosis-resistance genes, thereby significantly inhibiting ferroptosis [57]. However, we can see that this finding reveals an interesting paradox: LCN2 itself can induce ROS production, and ROS can activate ferroptosis; yet at the same time, it protects cells by activating antioxidant feedback loops.

In colorectal cancer (CRC), on the one hand, LCN2 inhibits ferroptosis by stimulating the expression of GPX4 and xCT, promoting tumor progression and the development of chemotherapy resistance [49]. On the other hand, Feng et al. demonstrated that LCN2 expression was reduced in Colorectal cancers (CRCs) harboring the BRAFV600E (V600E) mutation. Restoration of LCN2 expression could partially enhance intracellular iron accumulation, promote the iron-dependent Fenton reaction, increase ROS levels, and subsequently increase cisplatin vulnerability both *in vitro* and *in vivo* [58].

In acute lymphoblastic leukemia (ALL), the regulatory effect of LCN2 on ferroptosis differs markedly between adults and children. In childhood ALL, induction of LCN2 expression inhibits the growth of glucocorticoid-sensitive ALL cells and triggers ferroptosis [10]; In contrast, in T-cell acute lymphoblastic leukemia (T-ALL), silencing LCN2 enhances RSL3-induced ferroptosis [59]. These findings are likely associated with differences in leukemia subtype, experimental model, and therapeutic responses across age groups. Therefore the precise mechanisms remains to be determined.

**Figure 3:**
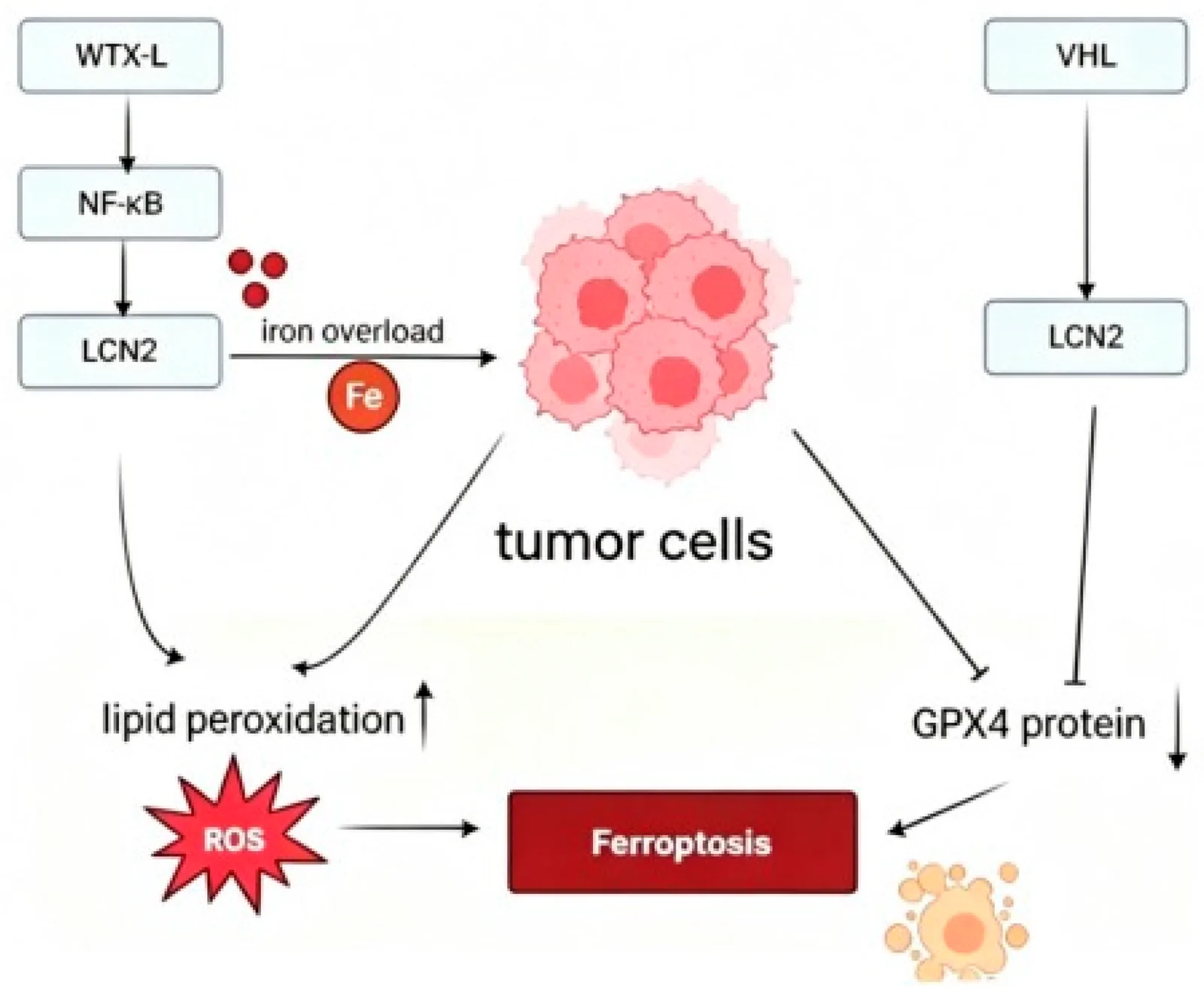
Special contexts where lipocalin 2 (LCN2) promotes ferroptosis: In gastric and renal cancer, LCN2 is upregulated, promoting ferroptosis by weakening the antioxidant system and increasing the production of reactive oxygen species. The figure was prepared using BioRender.com.

## 4 The Role of LCN2 on Iron in the TME

The TME is composed of tumor cells, stromal cells, and various immune cells, and is closely associated with tumor initiation and progression [60]. Iron metabolism reprogramming within the TME plays a critical role in tumor progression [21]; In the TME, persistent inflammation and increased secretion of siderophores by tumor cells and immune cells lead to dynamic changes in the ratio of apo-LCN2 to holo-LCN2, which in turn regulates iron metabolism in tumor cells [61]. At the same time, various immune cells can influence iron distribution in the microenvironment and the susceptibility of tumor cells to ferroptosis by secreting LCN2.

### 4.1 Cell-Derived LCN2 as Alternative Iron Supply

Previous studies have shown that macrophages play a significant role in tumor initiation and progression. In breast cancer models with high LCN2 expression, tumor-associated macrophages (TAMs) can actively secrete iron into the TME and, through iron uptake mediated by the LCN2 receptor (for example, 24p3R) on the surface of tumor cells, directly promote tumor cell proliferation, migration, and adhesion to the matrix [21]. *In vitro* co-culture experiments further confirm that the interaction between macrophages and cancer cells significantly upregulates LCN2 levels, promoting iron transport within the microenvironment and tumor proliferation [62]. Furthermore, the C5a-C5aR pathway of the complement system promotes the polarization of macrophages toward the M2 phenotype and upregulates LCN2 expression via endoplasmic reticulum stress, thereby enhancing iron transfer from macrophages to gastric cancer cells and accelerating gastric cancer progression [63]. Moreover, in a model of meningeal metastasis, macrophage-derived cytokines can induce LCN2 expression in cancer cells, enabling them to acquire iron from cerebrospinal fluid; this iron not only promotes the growth of cancer cells but also inhibit macrophage absorption and phagocytosis of iron [64].

In addition TAMs, in a mouse model of lung cancer-associated cachexia, Wang and colleagues showed that tissue-infiltrating neutrophils (TI-Neu) secrete large amounts of LCN2, inducing ferroptosis in adipose and muscle tissues and driving the development of cachexia [22].

In addition, recent research has revealed that cancer-associated fibroblasts (CAFs) in the TME regulate ferroptosis and immunotherapy resistance in NSCLC. LCN2 secreted by CAFs can be taken up by cancer cells, upregulating LCN2 levels, thereby inhibiting ferroptosis, reducing the release of damage-associated molecular patterns triggered by ferroptosis in tumor cells, and ultimately suppressing the antitumor immune response and mediating resistance to anti-PD-1 immunotherapy [65] (Fig. 4).

**Figure 4:**
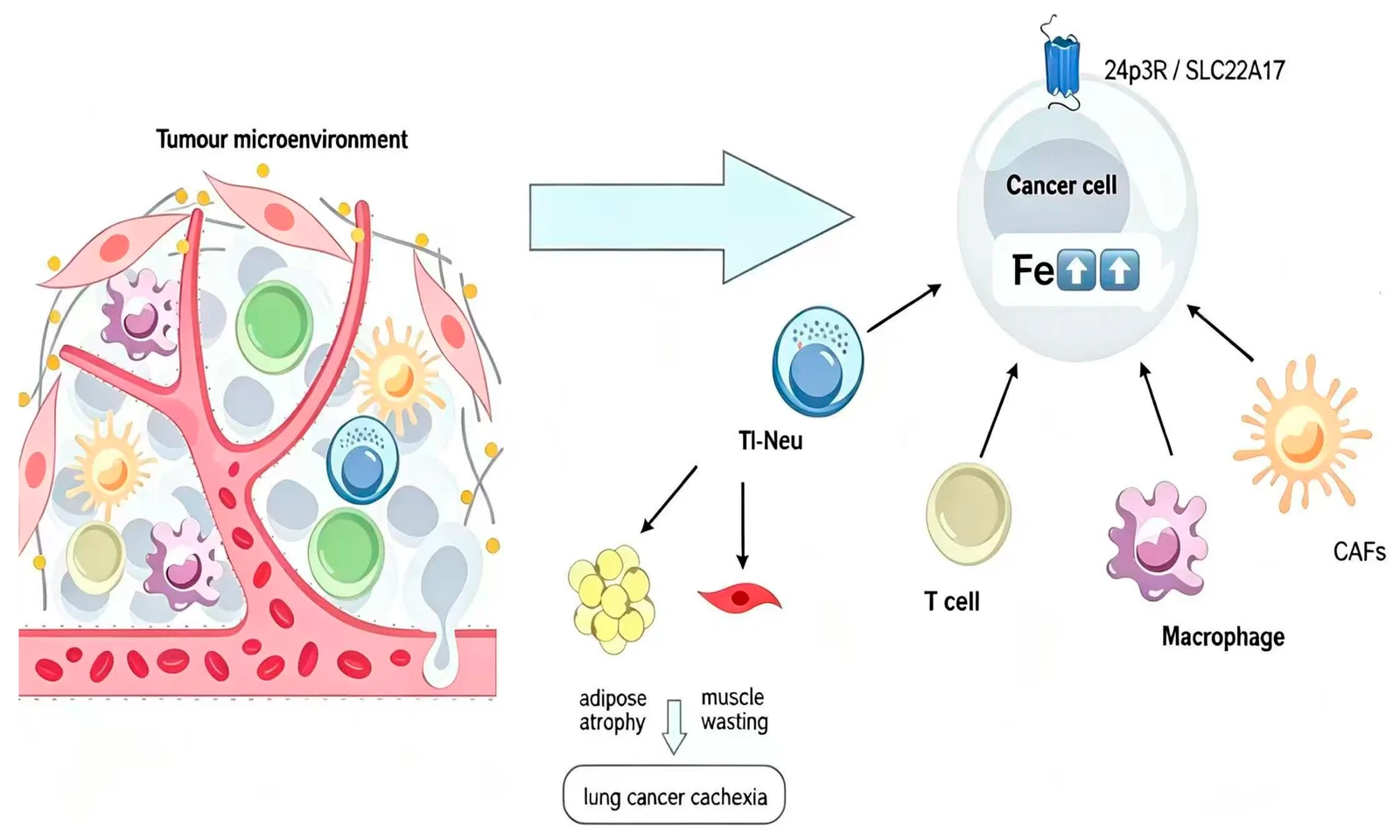
Cell-Derived lipocalin 2 (LCN2) in the tumor microenvironment: Macrophages, T cells, tissue-infiltrating neutrophils (TI-Neu), and cancer-associated fibroblasts (CAFs). All of them can deliver iron to tumor cells through LCN2, thereby promoting cancer cell proliferation. The figure was prepared using BioRender.com.

### 4.2 Hypoxia

A recent study has revealed a new mechanism by which the hypoxic microenvironment regulates ferroptosis in gastric cancer through LCN2: hypoxia significantly induces the expression of myeloid-derived growth factor (MYDGF), which maintains LCN2 protein stability by blocking endoplasmic reticulum-associated degradation (ERAD), thereby inhibiting ferroptosis in gastric cancer [66].

The TME plays a complex role in the occurrence and development of tumors, and the states of various cells and microenvironments can regulate iron metabolism through LCN2, thereby affecting tumor progression. Therefore, understanding the mechanisms involved can help to gain a deeper understanding of ferroptosis pathways and is crucial for developing more accurate and clinically feasible diagnostic strategies.

## 5 Targeted LCN2-Based Cancer Therapy Strategies

At present, a large number of studies have clearly shown that the LCN2 ferroptosis axis plays a key role in the pathogenesis of various diseases. Therefore, research on the regulation of LCN2 can become an effective approach for treating various ferroptosis related diseases.

### 5.1 Direct Targeting of LCN2

Currently, precise targeting strategies against LCN2 are under active investigation. Notably, LCN2 neutralizing antibodies have been preliminarily validated for efficacy in various cell and animal experiments. In patient-derived xenograft (PDX) models from HCC patients, the combination of LCN2 neutralizing antibodies and sorafenib significantly enhances sorafenib-induced ferroptosis and demonstrates superior tumor growth inhibition compared to monotherapy [67]. Similarly, in colorectal cancer, after treatment with LCN2 monoclonal antibody (3D12B2), the intracellular iron content increased and in combination with 5-FU, it can further inhibit tumor progression and induce tumor regression [49]. In a lung cancer cachexia model, LCN2 neutralizing antibodies reduce the wasting of adipose and muscle tissue and prolong survival in mice [22]. In addition to antibody-based approaches, various nanodelivery systems have been developed for LCN2 targeting: octreotide (OCT)-modified cationic liposomes encapsulating LCN2 siRNA can selectively target breast cancer cells via endocytosis mediated by the somatostatin receptor on the surface of cancer cells, thereby silencing LCN2 mRNA and significantly reducing tumor angiogenesis and endothelial cell migration [68]. In addition, Trastuzumab-conjugated liposomes loaded with LCN2 siRNA can be specifically internalized by HER2(+) IBC cells, significantly reducing LCN2 mRNA levels and disrupting tumor embolization [69]. Huang et al. found that in an LCN2-rich TME, E. coli engineered to efficiently absorb iron ions while avoiding binding to LCN2 significantly enhanced its antitumor efficacy in mice [31]. Iron oxide nanoparticles are a multifunctional nanocarrier that can serve as both an iron source and a drug delivery system. Iron oxide nanoparticles loaded with miR-214-3p can be delivered specifically to tumor tissues, where they simultaneously inhibit GPX4 and LCN2—two key ferroptosis-inhibiting molecules—significantly increasing intracellular iron concentrations and lipid peroxidation levels, thereby promoting ferroptosis [70].

Gene silencing or knockout of LCN2 is one of the effective strategies for restoring sensitivity to ferroptosis and suppressing tumors. siRNA-mediated LCN2 silencing has demonstrated significant antitumor effects in various mouse cancer models: Valashedi et al. found that CRISPR/Cas9-mediated LCN2 knockout in breast cancer cells significantly reduced cell proliferation and migration, promoted erastin-mediated ferroptosis, and increased sensitivity to cisplatin [71]; Additionally, Gao et al. developed a therapy called Gene Interference Ferroptosis Therapy (GIFT), which uses Cas13a or miRNA to specifically knockdown LCN2 and FPN in cancer cells while simultaneously administering iron nanoparticles [72].

LCN2 inhibitors represents a promising alternative strategy. For example, Santiago-Sánchez et al. have screened out small molecular compounds ZINC00640089 and ZINC00784494 through virtual screening, which have been identified as LCN2 interacting agents that can inhibit the proliferation of inflammatory breast cancer cells and reduce AKT phosphorylation levels [73].

It is worth noting that, as described in the introduction, validating effective therapeutic candidate drugs in mouse models may face completely different molecular environments in the human body. Future clinical studies should take into account this difference to better evaluate the efficacy and safety of LCN2 treatment before clinical translation.

### 5.2 Small-Molecule Inhibitors and Natural Products

In 2024, Sun et al. discovered that the small-molecule compound G-4 exerts an anti-triple-negative breast cancer effect by downregulating LCN2 levels, thereby promoting ferroptosis [74].

Various natural products exert antitumor effects by regulating the LCN2-ferroptosis axis. For example, polyphenols (such as curcumin) can inhibit the progression of hepatocellular carcinoma by modulating the LCN2-ferroptosis axis [75]. Rhizoma Paridis total saponins (RPTS) can induce ferroptosis by inhibiting the SPI1/LCN2 axis [76], offering a new strategy for the treatment of osteosarcoma.

However, translating these approaches into clinical applications remains challenging. Unlike classical oncogenic drivers, LCN2 also performs essential physiological functions, including innate immune defense, bacterial iron restriction, and tissue repair. Therefore, systemic inhibition of LCN2 may cause unintended effects, including impaired host defense or abnormal iron homeostasis. Future therapeutic development should prioritize tumor-selective delivery systems or transient inhibition strategies to maximize antitumor efficacy while minimizing toxicity.

## 6 Research Progress and Clinical Value of LCN2 as a Prognostic Biomarker

As a molecular protein associated with injury, the expression level of LCN2 significantly increases under conditions such as inflammation, hypoxia, and infection. Therefore, in most diseases, it becomes an early warning signal of tissue damage [77]. Currently, LCN2 has been extensively studied in various types of cancer, making it a promising diagnostic and prognostic biomarker.

For instance, LCN2 is one of the most extensively studied prognostic markers in breast cancer. Wei et al. found a significant increase in plasma LCN2 level in breast cancer patients, especially in patients with negative progesterone receptor, tissue grade ≥ 2, clinical stage III, or pathologic T2 + T3 + T4 [78]. Furthermore, Bauer et al. analyzed samples from 207 breast cancer patients and found that high LCN2 levels were inversely associated with ER and PR scores (*p* < 0.0001; *p* = 0.0003, respectively), The proportion of high-grade (grades ≥ 2) (*p* = 0.0218), HER-2/neu overexpression (*p* = 0.0018), lymph node metastases (*p* < 0.0001) and Ki-67 proliferation index (*p* < 0.0001) was significantly higher in patients with positive NGAL tumors [79]. Patients with elevated serum LCN2 and MMP-9 levels also had significantly shorter recurrence-free survival (*p* = 0.004), particularly among women with BMI less than 25 kg/m^2^ (*p* < 0.001) or whose breast tumors had not metastasized to the lymph nodes (*p* < 0.001) [80].

In the field of lung cancer, LCN2 is significantly upregulated in LUAD and positively correlated with clinical stage, tumor size, lymph node metastasis, and distant metastasis, showing high diagnostic efficiency. Its area under the curve (AUC) is 0.818, especially for stage III-IV patients, it can reach 0.895 [81].

In patients with liver metastases from colorectal cancer, adjusted serum NGAL levels (CNL) are significantly associated with more than three tumor nodules and disease involving both hepatic lobes, patients with high CNL levels have a poorer prognosis [82].

Additionally, in EC, the expression of LCN2 protein is associated with non endometrioid histological type, nuclear grade 3, tumor solid fraction exceeding 50%, estrogen receptor and progesterone receptor negativity, and EZH2 protein expression positivity. It is worth noting that LCN2 is also significantly associated with distant tumor recurrence. Patients with strong expression of LCN2 has only a 5-year survival rate of 38% [83].

## 7 Conclusions and Limitations

In this review, we synthesize that LCN2 has emerged as a critical and context-dependent regulator of ferroptosis in cancer. In the vast majority of solid malignancies, LCN2 functions as a protective factor that suppresses ferroptosis. This ferroptosis-resistant state promotes tumor progression, facilitates metastatic dissemination, and confers broad therapeutic resistance. Conversely, in distinct biological contexts, LCN2 can paradoxically function as a ferroptosis promoter.

Beyond its cell-autonomous functions, LCN2 serves as a critical intercellular messenger within the TME, iron is not merely a cellular metabolite but a shared resource that is continuously redistributed among malignant cells, immune populations, and stromal compartments. Tumor-associated macrophages and neutrophils can serve as important sources of extracellular LCN2, suggesting that LCN2 functions as an intercellular iron trafficking mediator. By regulating iron transfer between different cellular populations, LCN2 may reshape the metabolic landscape of the tumor ecosystem and determine which cells become ferroptosis-resistant or ferroptosis-sensitive. Thus, LCN2 should be considered not only as an intracellular regulator of ferroptosis but also as an extracellular coordinator of iron allocation within tumors.

Despite substantial advances, several fundamental questions remain unresolved. First, Future studies should systematically investigate how apo-LCN2 and holo-LCN2 differentially regulate iron trafficking, receptor activation, and downstream signaling pathways. Second, although LCN2 represents an attractive therapeutic target, its physiological functions create significant translational challenges. LCN2 contributes to host antimicrobial defense, inflammatory regulation, and tissue homeostasis; therefore, systemic inhibition may result in undesirable effects. Future therapeutic strategies should focus on tumor-selective targeting approaches. Finally, targeting the LCN2–ferroptosis axis may provide new opportunities for combination therapies. Because LCN2 contributes to ferroptosis resistance through multiple mechanisms, combining LCN2 inhibition with ferroptosis inducers, chemotherapy, radiotherapy, or immune checkpoint blockade may overcome therapeutic resistance and enhance antitumor efficacy. However, rational combination strategies will require a deeper understanding of tumor-specific ferroptotic dependencies and the spatial organization of iron metabolism within the tumor ecosystem.

Collectively, LCN2 plays an important role in ferroptosis. In depth analysis of its role not only helps to clarify the occurrence, development, and drug resistance mechanisms of tumors, but also utilizes its diagnostic and prognostic evaluation functions to provide guidance for the development of new treatment methods for ferroptosis. However, to achieve this, a deeper understanding of the interaction between LCN2 and tumor iron metabolism is needed, and these basic findings need to be translated into strategies that can effectively improve patient treatment and survival.

## Data Availability

Not applicable.
